# Plasma acylcarnitine concentrations reflect the acylcarnitine profile in cardiac tissues

**DOI:** 10.1038/s41598-017-17797-x

**Published:** 2017-12-13

**Authors:** Marina Makrecka-Kuka, Eduards Sevostjanovs, Karlis Vilks, Kristine Volska, Unigunde Antone, Janis Kuka, Elina Makarova, Osvalds Pugovics, Maija Dambrova, Edgars Liepinsh

**Affiliations:** 1 Latvian Institute of Organic Synthesis, Aizkraukles Str. 21, Riga, LV-1006 Latvia; 2 University of Latvia, Faculty of Biology, Jelgavas Str. 1, Riga, LV-1004 Latvia; 3 Riga Stradins University, Faculty of Pharmacy, Dzirciema Str. 16, Riga, LV-1007 Latvia

## Abstract

Increased plasma concentrations of acylcarnitines (ACs) are suggested as a marker of metabolism disorders. The aim of the present study was to clarify which tissues are responsible for changes in the AC pool in plasma. The concentrations of medium- and long-chain ACs were changing during the fed-fast cycle in rat heart, muscles and liver. After 60 min running exercise, AC content was increased in fasted mice muscles, but not in plasma or heart. After glucose bolus administration in fasted rats, the AC concentrations in plasma decreased after 30 min but then began to increase, while in the muscles and liver, the contents of medium- and long-chain ACs were unchanged or even increased. Only the heart showed a decrease in medium- and long-chain AC contents that was similar to that observed in plasma. In isolated rat heart, but not isolated-contracting mice muscles, the significant efflux of medium- and long-chain ACs was observed. The efflux was reduced by 40% after the addition of glucose and insulin to the perfusion solution. Overall, these results indicate that during fed-fast cycle shifting the heart determines the medium- and long-chain AC profile in plasma, due to a rapid response to the availability of circulating energy substrates.

## Introduction

Acylcarnitines (ACs) are formed from carnitine and acyl-CoAs by carnitine acyltransferases in mitochondria or peroxisomes^1,2^. ACs are generally considered to be a transport form of fatty acids (C2-C26) and can be utilized for energy production in mitochondria or for the synthesis of endogenous molecules; they can also be transported from tissues to plasma. An increase in plasma AC concentration has been linked to the progression of various diseases, including insulin resistance^3–7^ and cardiovascular diseases^8–12^. Since the metabolism of fatty acids, glucose and amino acids can yield ACs, their concentration in plasma is determined by the nutritional state and tissue- or organ-specific contributions. Therefore, to link a particular disease and nutrition state to the plasma AC profile, it is important to clarify the origin of ACs responsible for changes in the AC concentration in plasma.

The AC profile, or the concentrations of ACs with specific chain lengths, could characterize the energy metabolism pattern and could indicate the presence of fatty acid oxidation and organic acid metabolism disorders. In contrast to short-chain ACs that are produced from glucose, amino acids and fatty acid degradation, medium- and long-chain ACs are derived exclusively from fatty acid metabolism. Long-chain ACs are mainly synthesized and metabolized in mitochondria; therefore, concentrations of long-chain ACs are used as markers of mitochondrial fatty acid oxidation. Historically, an increased concentration of ACs has been used as a marker of incomplete fatty acid oxidation to diagnose inborn fatty acid oxidation defects^13^. However, an increased concentration of ACs is also observed in a fasted state when the fatty acid oxidation rate is increased^14–17^. Taking into account that long-chain fatty acids are mostly utilized by the heart and skeletal muscles^14,18,19^, changes in the concentrations of long-chain ACs in plasma should represent the fatty acid oxidation pattern of heart and skeletal muscle mitochondria.

Under physiological conditions, an organism has to switch between glucose and fatty acid metabolism based on the availability of substrates to maintain energy homeostasis. Circulating concentrations of substrates and hormones and concomitant changes in gene and protein expression determine the energy metabolism pattern in tissues^14,20–22^. It has been proposed that the plasma concentrations of ACs, particularly medium- and long-chain ACs, can predict the intracellular energy metabolism pattern and can be used as a marker of metabolic dysfunction^3–12,23,24^. However, in the studies that have suggested increased AC levels as a marker of insulin resistance, type 2 diabetes and cardiovascular diseases, the measurements were performed only in plasma or serum, and data regarding the AC content in tissues are lacking. To date, only a few studies have investigated the relationship between plasma and tissue AC contents^15–17,23,25,26^. Measurements of ACs in fed and fasted states showed that the plasma long-chain AC concentration reflects the AC content in cardiac tissue, but the data regarding the content in muscles are controversial^15–17,26^. These data indicate that plasma ACs relationship to the tissue ACs could be dependent on the metabolic condition imposed at the time of assessment. The time-dependent changes in tissue AC contents after glucose administration have never been investigated. It remains unclear how the plasma AC profile reflects the AC content in tissues after different stimulus (glucose administration or exercise) and which tissues are responsible for changes in the plasma AC profile. Thus, in the present study, we investigated the changes in the AC profile in plasma and tissues in the fed and fasted states, as well as time-dependent changes during an oral glucose tolerance test. In addition, the AC effluxes were measured in isolated organ models.

## Results

### Effects of fasting on AC concentrations in tissues

**Figure 1:**
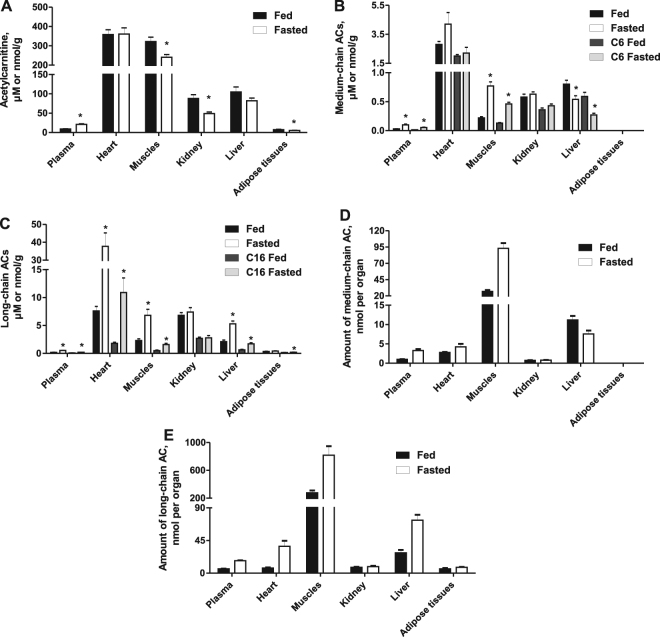
Acylcarnitine (AC) concentrations and amounts in plasma and different organs in the fed and fasted states. During fasting, the acetylcarnitine concentration was significantly increased in plasma but decreased in the skeletal muscles, kidney and adipose tissues (**A**). The medium-chain AC concentration was increased in plasma, heart and skeletal muscles and decreased in the liver (**B**) in fasted animals. The long-chain AC concentration was significantly higher in plasma, heart, skeletal muscles and liver in the fasted group. The highest amount of medium-chain (**D**) and long-chain (**E**) ACs per organ was observed in the heart, skeletal muscles and liver; moreover, the content of ACs in the plasma was comparable to the AC content in the heart. The results are presented as the average values ± SEM of 7 animals. *Indicates a significant difference compared with the fed group (Student’s t-test, P < 0.05).

As shown in Fig. 1, the highest AC content was observed in the heart (390–430 nmol/g), followed by muscles (250–350 nmol/g); the lowest contents were in plasma (10–25 µM) and in adipose tissues (6–8 nmol/g). Moreover, the highest amount of ACs per organ was found in the skeletal muscles, liver and heart (Fig. 1D,E).

Short-, medium- and long-chain AC concentrations were 2.1, 3.4, and 2.6 times higher, respectively, in plasma from overnight fasted animals than in plasma from fed animals (Fig. 1, Supplementary Table S2). In the heart, the content of medium-chain ACs was increased by only 53% (p = 0.09) (Fig. 1B), while an almost 5-fold higher content of long-chain ACs (Fig. 1C) was observed in the fasted state compared with the fed state samples. In skeletal muscle tissues, the acetylcarnitine content was 24% lower in the fasted animals than in the fed animals (Fig. 1A), while the medium- and long-chain AC contents in muscle were 3.5 and 2.9 times higher, respectively, in the fasted state compared with the fed state (Fig. 1B,C). In the liver, overnight fasting induced a 21–32% decrease in acetyl- and medium-chain AC contents (Fig. 1A,B), whereas a 2.5-fold increase in the long-chain AC content was observed (Fig. 1C). The acetylcarnitine content in epidydimal adipose tissues was 30% lower in the fasted state than in the fed state (Fig. 1A), while the long-chain AC content did not differ significantly between the fed and fasted states (Fig. 1C). Overnight fasting induced a decrease of 40% in the acetylcarnitine content in kidney tissues (Fig. 1A), whereas the medium- and long-chain AC contents were not changed (Fig. 1B,C). Interestingly, the amounts of medium- and long-chain ACs in plasma were comparable to the amount of ACs in the heart (Fig. 1D,E), indicating that changes in AC content in the heart could determine changes in the AC profile in plasma. Taken together, these results demonstrate that the most pronounced difference in AC concentration between the fed and fasted states was in tissues that exhibit the most pronounced metabolic flexibility, such as the heart, skeletal muscles and liver. Since changes in plasma AC concentrations, particularly medium- and long-chain ACs, reflected changes in AC content in these tissues, plasma AC concentrations could be a valid marker for AC tissue content.

### AC concentration during an oral glucose tolerance test (OGTT)

**Figure 2:**
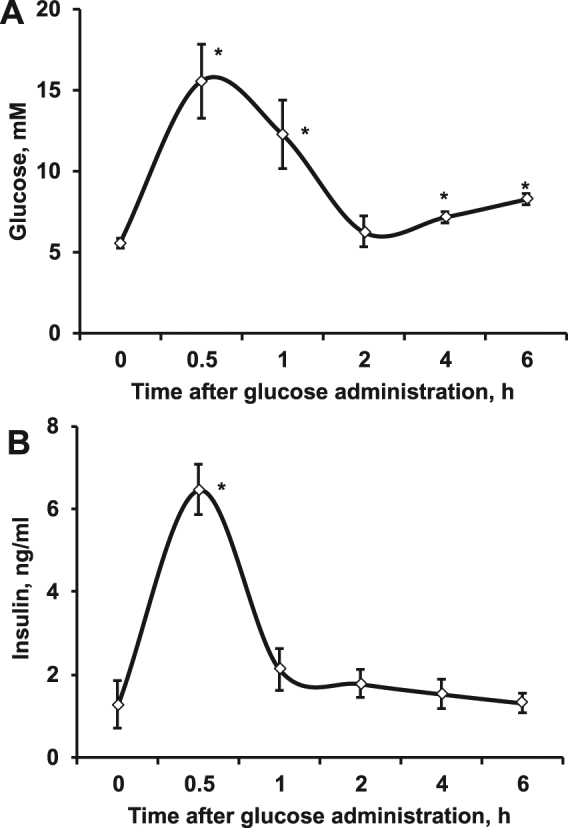
The concentrations of glucose and insulin in plasma during an oral glucose tolerance test. A significant increase in glucose (**A**) and insulin (**B**) concentration in plasma was observed after oral glucose administration. The results are presented as the average values ± SEM of 5 animals. *Indicates a significant difference compared with the baseline group (one-way ANOVA with Tukey’s post-test, P < 0.05).

**Figure 3:**
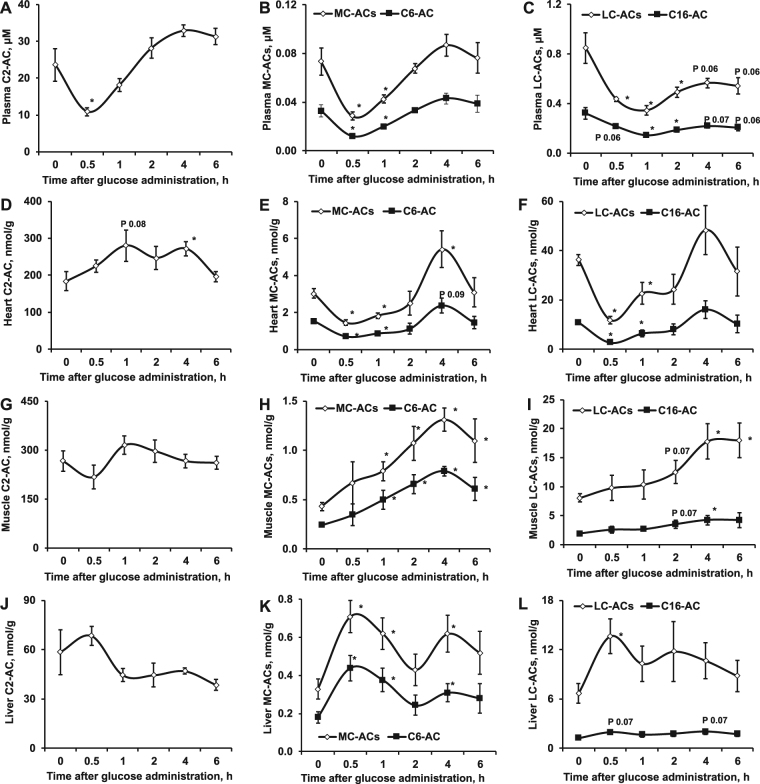
Acylcarnitine (AC) concentrations in plasma and different organs during an oral glucose tolerance test (OGTT). The concentrations of ACs (acetyl-(C2; **A**), medium- (MC; **B**) and long-(LC; **C**) chain) in plasma were decreased 30 min after glucose administration and then increased. The changes in the acetylcarnitine contents in the heart (**D**), skeletal muscles (**G**) and liver (**J**) were negligible during the OGTT. In the heart, 30 min after the administration of glucose, the contents of medium- (**E**) and long-chain (**F**) ACs were significantly decreased and slowly increased afterwards. After glucose administration, the contents of medium- (**H**) and long-chain (**I**) ACs increased in muscles, while in the liver, the contents of medium-(**K**) and long-chain (**L**) ACs varied over time. The results are presented as the average values ± SEM of 5 animals. *Indicates a significant difference from the baseline (one-way ANOVA with Tukey’s post-test, P < 0.05).

To evaluate whether the plasma AC profile can be used as a marker of AC content in tissues, the AC content was measured during an OGTT in the heart, hindlimb muscles and liver, and the possible association of plasma AC concentrations with AC content in these tissues was studied. During the OGTT, a significant increase in glucose and insulin concentrations was observed 30 min after glucose administration, followed by a rapid decrease in glucose and insulin concentrations almost to the baseline level (Fig. 2A,B). In contrast to glucose and insulin concentrations, the levels of all ACs in plasma were decreased 30 min after glucose administration and then gradually increased (Fig. 3A–C, Supplementary Table S3).

**Figure 4:**
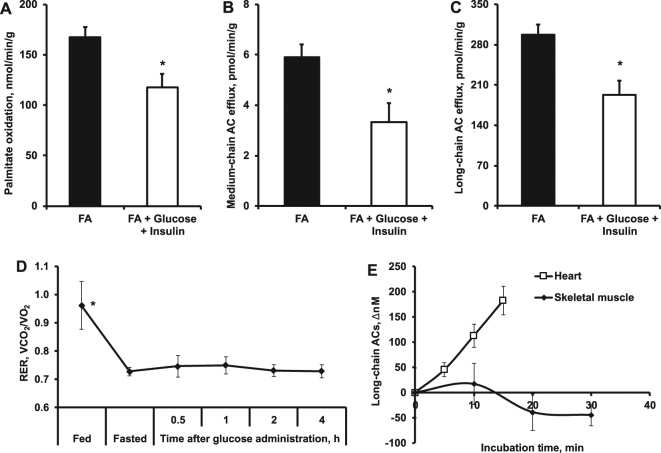
The energy metabolism pattern in the heart and muscles. In isolated rat hearts, the addition of glucose and insulin to a fatty acid (FA) perfusion solution significantly decreased palmitate oxidation (**A**) and medium- (**B**) and long-chain (**C**) AC efflux from tissues. Indirect calorimetry measurements (**D**) showed that after glucose administration, there were no changes in RER (0.7), indicating that muscles cannot rapidly switch from fatty acid to glucose metabolism. Time-dependent changes in long-chain AC concentration in buffer show that, in contrast to heart, in isolated mice contracting skeletal muscle model there is no significant efflux of long-chain ACs (**E**). The results are presented as the average values ± SEM of 6 hearts (**A**–**C**) or 5(**D**) or 4 (**E**) mice. *Indicates a significant difference from the control conditions (A – FA buffer; B – fasted state) (Student’s t-test or one-way ANOVA with Tukey’s post-test, P < 0.05).

Since short-chain ACs are of mixed origin (glucose, amino acid and fatty acid metabolism), the changes in their contents in tested tissues were negligible during the OGTT (Fig. 3D,G,J, Supplementary Tables S4–6). In the heart, 30 min after the administration of glucose, the contents of medium- and long-chain ACs were significantly decreased and slowly increased afterwards (Fig. 3E,F, Supplementary Table S4). No such changes in skeletal muscles and liver were observed (Fig. 3H,I,K,L), suggesting that the concentration of medium- and long-chain ACs in plasma reflects the changes in AC content in the heart. This was further confirmed by measurements of palmitate oxidation and long-chain AC efflux in the heart. In isolated rat hearts, the addition of glucose and insulin to the perfusion solution decreased palmitate oxidation by 30% (Fig. 4A) and decreased the medium- and long-chain AC efflux from tissues by 43% and 35%, respectively (Fig. 4B,C). These results demonstrate that the heart is able to react immediately to the changes in circulating substrates and switch its energy metabolism from fatty acid oxidation to glucose oxidation. Accordingly, the medium- and long-chain AC profile in the heart reflects changes in energy metabolism. Moreover, these results indicate that the heart could determine changes in plasma medium- and long-chain AC concentrations during the transition from fasted to fed state.

In contrast to the heart, the skeletal muscles did not respond to short-term changes in plasma glucose and insulin concentration. The fatty acid metabolism and corresponding contents of medium- and long-chain ACs were not changed during the OGTT (Fig. 3H,I, Supplementary Table S5). This fact was supported by an indirect calorimetry experiment in mice. The RER remained decreased (0.7) after glucose administration (Fig. 4D), indicating that skeletal muscles cannot rapidly switch from fatty acid to glucose metabolism. Moreover, in contrast to heart, in mice isolated skeletal muscle model, we did not observe any efflux of medium- (data not shown) and long-chain ACs (Fig. 4E), indicating that even the contracting skeletal muscles do not contribute to plasma AC pool.

In liver tissues, a significant increase in medium- and long-chain AC content was observed 30 min after glucose administration (Fig. 3K,L, Supplementary Table S6). Changes in medium- and long-chain AC contents in the liver (Fig. 3K,L) that did not correspond to changes in plasma AC content (Fig. 3B,C) were observed afterwards, indicating that the liver does not determine the changes in the plasma medium- and long-chain AC profile observed during the OGTT.

### AC concentrations in plasma, skeletal muscles and heart after exercise

**Figure 5:**
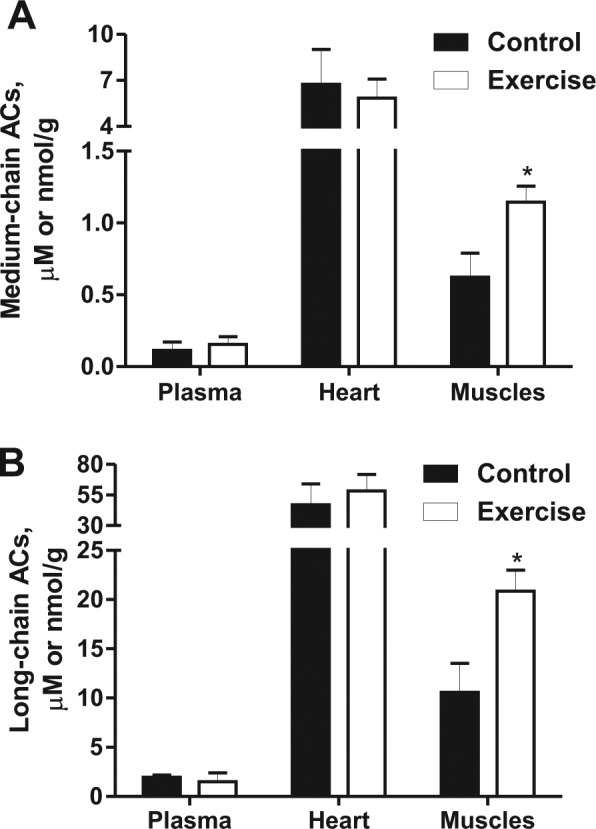
The medium- (**A**) and long-chain (**B**) acylcarnitine (AC) content in plasma, heart and skeletal muscle tissues after exercise. The exercise induced significant increase in medium- and long-chain AC content in muscles, while AC concentration in plasma and heart was not changed. The results are presented as the average values ± SEM of 6-7 animals. *Indicates a significant difference compared with the control group (Student’s t-test, P < 0.05).

Since skeletal muscles contain high amount of medium- and long-chain ACs and could contribute to plasma AC pool under conditions of the increased energy demand, we determined AC profile in plasma, skeletal muscles and heart after moderate intensity exercise. As seen in Fig. 5, after exercise, the medium- and long-chain AC contents in skeletal muscles were increased 1.8 and 2 times, respectively, compared to AC content in sedentary control group; however, no difference in medium- and long-chain AC concentrations was found in heart and plasma. These results support observation that changes in plasma medium- and long-chain ACs do not reflect changes in AC profile in skeletal muscles.

## Discussion

In the present study, we demonstrated that the most pronounced difference in AC content between the fed and fasted states was observed in tissues that are metabolically flexible. In addition, we showed that the amount of ACs in plasma is comparable with the heart tissue content of long- and medium-chain ACs and it can be easily affected by a metabolic switch from fatty acid to glucose oxidation in the heart. Moreover, the observed simultaneous changes in medium- and long-chain AC content both in plasma and in the heart after glucose administration indicate that plasma medium- and long-chain AC concentrations reflect the content of ACs found in the heart.

**Figure 6:**
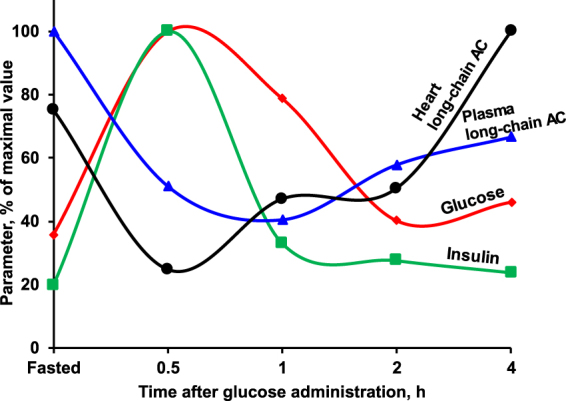
The time-dependent changes in glucose, insulin and long-chain acylcarnitine (AC) concentrations in plasma and in the long-chain AC content in the heart after glucose administration. The administration of glucose induces an increase in glucose (red line) and insulin (green line) concentrations and a simultaneous decrease in the long-chain AC concentration in the heart (black line) and plasma (blue line), indicating that the heart switches to glucose metabolism for energy production. Afterwards, when plasma glucose and insulin concentrations decrease, an increase in long-chain AC concentrations in the heart and plasma is observed, indicating that the heart switches back to fatty acid oxidation.

During fasting, the heart produces energy almost exclusively from fatty acid oxidation^14,18^; thus, a substantial increase in long-chain AC content is observed. Since the heart has limited stores of energy substrates^27,28^, it rapidly switches to the available circulating substrates. Thus, it is not surprising that immediately after glucose administration, a decrease in long- and medium-chain AC contents in the heart is observed as a result of the energy metabolism switch from fatty acid to glucose oxidation. Later, when the plasma glucose concentration decreases, the heart switches back to fatty acid oxidation, which results in increased cardiac contents of medium- and long-chain ACs (Fig. 6).

The energy metabolism pattern in skeletal muscles is similar to that in the heart. Skeletal muscles oxidize both fatty acid and glucose, and fatty acid metabolism is predominant when glucose reserves are depleted^19,29^. A corresponding increase in skeletal muscle AC content is also observed in the case of prolonged fasting^17^. During the OGTT, skeletal muscles did not react to glucose administration, while we observed a gradual increase in long-chain AC content 4–6 h after the OGTT. Since long-chain ACs play an active role in the regulation of energy metabolism and determine the metabolic pattern in muscles and the heart^15,23,30,31^, the increase in long-chain AC content during prolonged fasting hinders muscles from immediately switching to glucose metabolism; this metabolic state is described as transient postprandial insulin resistance^17,32,33^. In addition, the exercise-induced increase in muscle medium- and long-chain AC content did not induce any changes in concentrations of these ACs in plasma. In skeletal muscles long-chain AC content is 10–20-fold higher than in plasma; this difference should be sufficient to ensure efflux of long-chain ACs. It could be assumed that in case of any efflux of long-chain ACs from muscles, plasma AC concentration is maintained by increased uptake of ACs by other organs. However, in line with previous studies in humans^34^ and porcine model^35^, we did not observe any significant long-chain AC efflux from isolated mice contracting skeletal muscle. Overall, data suggest that there is negligible long-chain AC efflux from muscles under physiological conditions. Taking together these results show that the concentration of medium- and long-chain ACs in plasma does not reflect the muscle AC content shortly after glucose administration or after exercise.

Several studies have investigated the role of the liver in the regulation of the plasma AC pool^34,35^. It has been suggested that the increased level of long-chain ACs observed in the liver during fasting reflects an increase in fatty acid activation and subsequent oxidation^16^. In our study, we observed that fasting induces an increase in the liver content of long-chain ACs, but not medium-chain ACs, indicating that changes in the AC profile characterize the uptake rate of fatty acids and their metabolites rather than fatty acid oxidation in the liver. This is in line with previous observations of long-chain AC uptake by the liver in humans during overnight fasting^34^. Moreover, a recent study in liver-specific carnitine palmitoyltransferase 2 knock-out mice demonstrated that despite a dramatic increase in the long-chain AC content in the liver, the concentration of long-chain ACs in plasma was not affected^36^. Thus, it seems that the liver actively takes up and accumulates long- and medium-chain ACs rather than releasing them into the plasma.

It has been suggested that short-chain ACs in plasma are released from the liver, while medium-chain ACs are released from the liver and muscles^34,35^. Since in the heart, fatty acids are the preferred substrate for ATP production^14,18,27^, it has been suggested that long-chain ACs may be released from the heart^34^; however, no evidence for this has ever been provided. In the present study, we demonstrated that changes in the long-chain AC concentration in plasma during an OGTT reflect changes in long-chain ACs in the heart, but not in muscle or the liver. In addition, the medium- and long-chain AC efflux from the heart, but not from skeletal muscles, was observed *ex vivo*. Moreover, the amount of medium- and long-chain ACs in the heart is comparable to the amount of ACs found in plasma. Taken together, these data indicate that changes in the long-chain AC content in the heart can determine changes in the medium- and long-chain AC profile observed in plasma (Fig. 6) and that the heart is the major contributor to the long-chain AC pool in plasma.

The ability to switch between available energy substrates is crucial for cell functioning. Impaired metabolic flexibility is involved in the development of metabolic disorders such as insulin resistance and heart failure^20,21,37^. It has been demonstrated that the accumulation of lipid intermediates (diacylglycerols, ceramides, acyl-CoA, etc.) in the liver, skeletal muscle and heart induces metabolic inflexibility caused by mitochondrial dysfunction, activates inflammation pathways and impairs insulin signaling^19,38,39^. Though for decades ACs were thought to be only a transport form of acyl-moieties into mitochondria, recent studies have elucidated the active role of ACs in the regulation of energy metabolism and proposed AC accumulation as a link to insulin resistance^15,26,30,31^. Therefore, plasma AC concentration changes could be a valuable diagnostic marker for insulin resistance.

The potential limitations of our study is that OGTT characterizes the organ response to insulin stimulus induced by glucose administration, but not overall metabolic flexibility as transition from fasted to fed state. The OGTT is common method used in clinics to diagnose insulin resistance in peripheral tissues. Our data indicate, that addition of AC profile measurements in plasma during OGTT could be used to evaluate organ, particularly heart, specific insulin resistance; however, further studies are needed to investigate the potential application of AC profile measurements in the diagnostics of tissue specific insulin resistance.

A disturbed plasma AC profile is used as a marker for incomplete fatty acid oxidation to diagnose inherited disorders and metabolic inflexibility^4,13,24^. Only a few studies have investigated the association between the plasma AC profile and tissue AC concentrations^16,17^. In line with previous studies^16,17^, in the present study no association between the plasma AC profile and the AC profile in muscles and liver was observed during OGTT or after exercise. Nonetheless, the concentration of medium- and long-chain ACs in plasma reflect the AC profile in the heart, probably due to rapid energy turnover in the heart. Compared with other organs, the heart has the highest content of medium- and long-chain ACs. The content of medium- and long-chain ACs in the heart in the fed state is higher than that in other tissues in the fasted state. Thus, if AC flux from tissues to plasma depends on intracellular concentration, the highest possible efflux of medium- and long-chain ACs to plasma could be from the heart. In addition, AC content-related flux changes might be non-linear. This could explain why, despite being relatively small, changes in the medium- and long-chain AC content in the heart during a fed-fast cycle can significantly contribute to the plasma AC pool. Notably, in patients with heart failure, which is characterized by cardiac metabolic inflexibility, elevated concentrations of circulating long-chain ACs are associated with impaired cardiorespiratory capacity and an increased risk of adverse clinical outcomes^8^. Thus, the plasma medium- and long-chain AC concentration can be used as a marker of the cardiac energy metabolism pattern (Fig. 6) and the risk of cardiovascular diseases.

In conclusion, our results demonstrate that the heart is a major contributor to plasma AC concentration changes, particularly medium- and long-chain ACs. Moreover, the plasma AC profile can be used as an indicator of metabolic flexibility in cardiac tissue.

## Methods

### Animals and treatments

Fifty-six male Wistar rats weighing 320–350 g and 21 male CD1 mice (35–38 g) were obtained from the Harlan Laboratories BV (Netherlands) and adapted for two weeks prior to the experiments. All animals were housed under standard conditions (21–23 °C, 12 h light/dark cycle, relative humidity 45–65%) with unlimited access to food (R70 diet, Lactamin AB, Kimstad, Sweden) and water. To avoid anaesthesia effect on AC profile in tissues^40^, for tissue sample collection animals were sacrificed by decapitation. The experimental procedures were performed in accordance with the guidelines of the European Community as well as local laws and policies, and the procedures were approved by the Latvian Animal Protection Ethical Committee of the Food and Veterinary Service, Riga, Latvia. All studies involving animals were reported in accordance with the ARRIVE guidelines^41,42^.

Animals were randomly separated into two experimental groups, fed (n = 7) and fasted (n = 7). The rats in the fed group had unlimited access to food, whereas those in the fasted group were deprived of food for 18 h prior to the start of the experiment. Fasting was started at the end of the light phase. The animals were sacrificed, and samples of plasma and heart, hindlimb muscle, liver, kidney and epidydimal adipose tissues were collected. Thirty additional rats were used for the oral glucose tolerance test (OGTT) experiment (n = 5 per time point). The rats were fasted overnight for 18 h, and then a glucose solution (0.5 g/kg of body weight) was administered *per os*. Samples (plasma and heart, hindlimb muscle and liver tissues) were collected 0, 0.5, 1, 2, 4 and 6 h after glucose administration. For the exercise experiment, 21 wheels forced exercise/walking wheel apparatus (PsymCon Model 35500, Lafayette Instrument, Lafayette, USA) was used. Before the experiment, mice (n = 14) were adapted to exercise for one week: on the first and second day mice walked 30 min at a speed of 3 m/min, on the third day they walked 40 min at a speed of 3.5 m/min, on the fourth day they walked 50 min at a speed of 4 m/min and on the fifth day they walked 60 min at a speed of 5 m/min. Mice were fasted overnight for 18 h. To ensure low AC concentration in plasma and high AC content in skeletal muscles that could enhance AC transport from muscles to plasma, before the experiment glucose at a dose of 0.3 g/kg was administrated intraperitoneally. After 30 min, mice (n = 7) were placed in the forced running wheel and were running 1 h at a speed of 10 m/min. Other 7 mice were used as a sedentary control group. After the experiment, plasma, heart and hindlimb muscles were collected. All samples were stored at −80 °C until analysis. An additional 12 rats were used for the isolated heart experiment. Five mice were used for the indirect calorimetry assay. An additional 4 mice were used for the isolated skeletal muscle experiment.

### Determination of the AC profile in plasma and tissues

The AC contents in the rat plasma and tissue samples were measured using ultra-performance liquid chromatography-tandem mass spectrometry (UPLC-MS-MS) as described by Kivilompolo *et al*.^43^ with some modifications. The tissue extraction was performed as previously described^44^ with modifications. Briefly, 0.4 ml of freshly prepared 100 mM potassium phosphate monobasic (KH_2_PO_4_, pH 4.9) and 1 ml of acetonitrile/2-propanol/methanol 3:1:1 (v/v) was added to 100 mg of tissue or 100 µl of plasma. The sample was sonicated for 30 s and centrifuged at 16000 g for 10 min. The supernatant was used for UPLC MS/MS analysis. A Waters Acquity UPLC H-Class chromatograph was coupled to a Waters Xevo TQ-S tandem mass spectrometer. Chromatographic separation was performed on a Waters Acquity UPLC BEH Hilic (2.1 × 100 mm, 1.7 µm) column in gradient mode. Solvent A was 10 mM ammonium acetate with 0.2% formic acid in water, and solvent B was acetonitrile. The initial mobile phase composition was 10% solvent A and was linearly increased to 20% solvent A over 7 minutes. The total run time with column flush and re-equilibration was 10 minutes. The column temperature was 30 °C, and the flow rate was 0.5 ml/min. Data acquisition was performed in positive electrospray ionization (ESI+) and multiple reaction monitoring (MRM) mode. The ion source parameters were as follows: source temperature, 120 °C; capillary voltage, 2.5 kV; desolvation gas temperature, 600 °C; desolvation and cone gas flow, 800 L/h and 150 L/h, respectively. The MRM transitions with cone and collision energy values are presented in Supplementary Table S1. The concentrations of ACs were measured against a nine-point standard curve of C4-C18 and C2-C3 ACs within a range of 0.01 nM to 20 nM and 0.2 nM to 200 nM in an analytical sample, respectively. The derivates with carbon chain C2-C5, C6-C12, C14-C18 were considered as short-, medium- and long-chain ACs, respectively. The concentration of ACs was expressed µM for plasma and nmol per mg of wet weight for tissues.

In addition, the amount of ACs per organ was calculated taking into account the average weight of whole organ or plasma volume^45,46^: concentration (nmol/g or µM) × organ weight or plasma volume.

### Determination of the glucose and insulin concentrations in plasma

The plasma glucose and insulin concentrations were determined using a kit from Instrumentation Laboratory and a Rat/Mouse Insulin ELISA kit (Millipore, Billerica, USA).

### Determination of the energy metabolism pattern in heart and muscles

The rate of radiolabeled palmitate oxidation was measured in fasted Wistar rat hearts as previously described^14^ with the modifications indicated below. Briefly, rats were anesthetized using sodium pentobarbital (60 mg/kg i.p.) with the concomitant administration of heparin (1000 IU/kg). The hearts were excised and retrogradely perfused with the respective oxygenated (95% O_2_ - 5% CO_2_) perfusion solutions via the aorta at a constant pressure of 70 mm Hg. The hearts were perfused with the respective non-labeled oxygenated Krebs-Henseleit (KH) buffer solution (118 mM NaCl, 4.7 mM KCl, 2.52 mM CaCl_2_, 1.64 mM MgCl_2_, 24.88 mM NaHCO_3_, 1.18 mM KH_2_PO_4_ and 0.05 mM EDTA, pH 7.4 at 37 °C) supplemented with palmitate, glucose and insulin for 30 min. Fatty acid buffer was used to mimic the fasted state and contained 0.8 mM sodium palmitate bound to 2% BSA, 5 mM glucose, and 0.3 ng/ml insulin. To study the transition from fasted to fed state, fatty acid buffer was supplemented with additional glucose and insulin at final concentrations of 10 mM and 3 ng/ml, respectively. After 1 h of perfusion, the solution was then switched to the respective oxygenated radiolabeled KH buffer solution for 10 min. Palmitate oxidation was determined by measuring ^3^H_2_O released from [9,10-^3^H]palmitate (specific activity, 60 Ci/mmol). In addition, the AC profile was measured in the collected perfusate to determine the AC efflux from heart tissues.

Since skeletal muscle metabolism is the major determinant of whole-body energy expenditure^47,48^, the skeletal muscle energy metabolism pattern was determined using indirect gas calorimetry. The PhenoMaster system for mice (TSE, Germany), with automated food/liquid access control units and an indirect gas calorimetry system, was used to monitor 24 h changes in energy metabolism. The mice were adapted to the PhenoMaster system for 72 h before the experiment. Adapted mice were fasted overnight, and glucose (0.5 mg/kg i.p.) was then administered. The rates of oxygen consumption (VO_2_) and carbon dioxide production (VCO_2_) were monitored to estimate the respiratory exchange rate (RER), an indicator of the energy metabolism pattern in skeletal muscles.

For isolated muscle experiment, mice fasted overnight were euthanized by cervical dislocation. Hindlimbs of the animal were sprayed with 70° ethanol and pinned on a support board. The skin was cut through the entire length of the limb and the underlying muscles were exposed. Fascia and tibialis anterior muscle were carefully removed and extensor digitorum longus muscle was exposed. A loop of surgical silk thread was tied around the distal tendon of the muscle and a platin hook was tied to the proximal end of the muscle using surgical thread. Both tendons were cut and muscles were transferred to ice-cold KH buffer solution. Further the muscles were attached to a force transducer and positioned between two platinum rings so that the muscle could be stimulated electrically and the resultant force response recorded. Muscles were stretched to an optimal level and stimulated with supra-maximal voltage. The experiment was performed using KH buffer solution supplemented with 0.8 mM sodium palmitate bound to 2% BSA, 5 mM glucose, and 0.3 ng/ml insulin. Muscles were allowed to adapt to for 30 min, then every 10 min for 30 min the buffer solution was collected to determine the AC efflux from muscle tissues.

### Statistical analysis

All data are expressed as the mean ± standard error of the mean (SEM). For statistical analysis, Student’s t-test or a one-way ANOVA with Tukey’s post-test were used. P values less than 0.05 were considered to be statistically significant. The sample size was not sufficient to perform statistical correlation analysis. Statistical calculations were performed using Prism 5.03 software (GraphPad, San Diego, California).

### Data availability

The datasets generated during and analysed during the current study are available from the corresponding author on reasonable request.

## Electronic supplementary material

Supplementary information
