# Supplementary material for: Plasma acylcarnitine concentrations reflect the acylcarnitine profile in cardiac tissues
Source: Sci Rep. 2017 Dec 13;7:17528. doi: 10.1038/s41598-017-17797-x (PMC5727517; doi:10.1038/s41598-017-17797-x)
Supplement: Supplementary file 1 — Supplementary information [file 41598_2017_17797_MOESM1_ESM.pdf]

Plasma acylcarnitine concentrations reflect the acylcarnitine profile in cardiac tissues

Marina Makrecka-Kuka[1,*], Eduards Sevostjanovs[1], Karlis Vilks[1,2], Kristine Volska[1,3], Unigunde Antone[1], Janis Kuka[1], Elina Makarova[1], Osvalds Pugovics[1], Maija Dambrova[1,3], Edgars Liepinsh[1]

[1] Latvian Institute of Organic Synthesis, Aizkraukles Str. 21, Riga, LV-1006, Latvia
[2] University of Latvia, Faculty of Biology, Jelgavas Str 1, Riga, LV-1004, Latvia
[3] Riga Stradins University, Faculty of Pharmacy, Dzirciema Str. 16, Riga, LV-1007, Latvia

Supplementary material

Supplementary Methods

Determination of the AC profile in plasma and tissues

Supplementary table S1. MRM parameters for acylcarnitine determination

Acylcarnitine	Transition, m/z	Cone voltage, V	Collision energy
C2	204.2→84.9	32	18
C3	218.2→84.9	30	18
C4	232.2→84.9	30	20
C5	246.2→84.9	30	20
C6	260.3→84.9	30	20
C8	288.3→84.9	30	20
C10	316.3→84.9	30	20
C12	344.3→84.9	30	20
C14	372.4→84.9	30	22
C16	400.5→84.9	30	26
C18:0	428.5→84.9	30	26
C18:1	426.4→84.9	30	26
C18:2	424.4→84.9	30	26

Supplementary Results

Supplementary table S2 Acylcarnitine (AC) concentrations in plasma and different organs in the fed and fasted states.

	**Plasma, µM**		**Heart, nmol/g**		**Muscles, nmol/g**		**Kidney, nmol/g**		**Liver, nmol/g**		**Adipose tissues, nmol/g**	
	**Fed**	**Fasted**	**Fed**	**Fasted**	**Fed**	**Fasted**	**Fed**	**Fasted**	**Fed**	**Fasted**	**Fed**	**Fasted**
**C2**	9±1	21±1*	358±25	361±32	322±23	241±14*	88±10	49±4*	105±14	82±7	7.4±0.8	5.2±0.5*
**C3**	0.38±0.01	0.23±0.02*	9.8±2.7	9.9±0.8	4.5±0.4	4.5±0.5	9.3±0.8	7.2±0.7*	3.2±0.3	6.6±0.6*	0.20±0.04	0.11±0.02
**C4**	0.95±0.08	0.29±0.02	17.9±2	19.8±0.6	3±0.3	4.6±0.1*	7.5±0.6	5.3±0.3*	11.3±1.3	2.7±0.2*	0.25±0.03	0.10±0.01*
**C5**	0.10±0.01	0.10±0.01	1.3±0.2	1.4±0.2	0.7±0.1	0.4±0.04*	3±0.2	2.2±0.1*	0.66±0.03	0.68±0.05	0.049±0.006	0.045±0.004
**C6**	0.015±0.001	0.055±0.006*	2±0.1	2.2±0.4	0.1±0.01	0.5±0.03*	0.4±0.03	0.4±0.03	0.59±0.07	0.27±0.03*	ND	ND
**C8**	0.005±0.000	0.015±0.002*	0.4±0.03	0.7±0.1*	0.04±0.005	0.1±0.01*	0.1±0.01	0.1±0.01	0.13±0.01	0.12±0.01	ND	ND
**C10**	0.005±0.000	0.015±0.002*	0.2±0.02	0.6±0.2*	0.03±0.005	0.1±0.01*	0.038±0.005	0.026±0.008	0.06±0.01	0.08±0.01	ND	ND
**C12**	0.005±0.000	0.015±0.001*	0.2±0.03	0.6±0.1*	0.03±0.005	0.1±0.01*	0.071±0.016	0.054±0.015	0.02±0.01	0.07±0.01*	ND	ND
**C14**	0.012±0.000	0.023±0.002*	0.3±0.04	1.5±0.4*	0.1±0.005	0.2±0.02*	0.20±0.03	0.21±0.05	0.07±0.02	0.13±0.02*	0.012±0.004	0.014±0.003
**C16**	0.09±0.01	0.21±0.01*	1.8±0.2	10.9±2.6*	0.5±0.1	1.6±0.2*	2.7±0.2	2.8±0.4	0.6±0.1	1.7±0.2*	0.15±0.02	0.22±0.02*
**C18:0**	0.018±0.001	0.042±0.003*	1.8±0.1	5.7±0.8*	0.3±0.02	0.6±0.1*	1.3±0.1	1.6±0.1	0.25±0.02	0.85±0.08*	0.042±0.01	0.077±0.004*
**C18:1**	0.074±0.001	0.214±0.010*	3.3±0.4	16.5±3*	1.3±0.2	3.9±0.7*	2.2±0.2	2.3±0.3	1.0±0.1	2.2±0.2*	0.14±0.02	0.12±0.01
**C18:2**	0.006±0.000	0.035±0.002*	0.5±0.1	3.1±0.8*	0.2±0.02	0.5±0.1*	0.41±0.03	0.35±0.02	0.13±0.02	0.38±0.03*	ND	ND

The results are presented as the average values ± SEM of 7 animals. * indicates a significant difference compared with the fed group (Student’s t-test, $P < 0.05$).

Supplementary table S3 Acylcarnitine (AC) concentrations in plasma during an oral glucose tolerance test.

	**Time after glucose administration**					
µM	**0 h**	**0.5 h**	**1 h**	**2 h**	**4 h**	**6 h**
**C2**	23.6±4.4	10.9±1.1*	18.2±1.7	28.2±2.8	32.9±1.6	31.3±2.2
**C3**	0.19±0.03	0.21±0.02	0.21±0.03	0.24±0.02	0.24±0.03	0.20±0.02
**C4**	0.23±0.04	0.20±0.03	0.28±0.02	0.36±0.03*	0.35±0.02*	0.30±0.03
**C5**	0.09 ±0.02	0.07±0.01	0.07±0.004	0.10 ±0.01	0.09±0.01	0.09±0.01
**C6**	0.03±0.005	0.01±0.002*	0.02±0.002*	0.03±0.002	0.04±0.004	0.04±0.01
**C8**	0.010±0.001	0.005±0.001*	0.007±0.001*	0.011±0.001	0.013±0.001	0.011±0.002
**C10**	0.009±0.001	0.004±0.000*	0.006±0.000*	0.009±0.001	0.011±0.001	0.009±0.001
**C12**	0.021±0.004	0.007±0.001*	0.010±0.001*	0.015±0.002	0.020±0.002	0.017±0.003
**C14**	0.09±0.02	0.03±0.003*	0.03±0.003*	0.05±0.01*	0.06±0.01	0.06±0.01
**C16**	0.32±0.05	0.22±0.01*	0.14±0.01*	0.19±0.02*	0.22±0.01*	0.21±0.02*
**C18:0**	0.061±0.008	0.054±0.002	0.041±0.004*	0.043±0.005	0.050±0.002	0.049±0.006
**C18:1**	0.33 ±0.05	0.12±0.01*	0.12±0.02*	0.19±0.01*	0.21±0.01*	0.20±0.02*
**C18:2**	0.045±0.006	0.012±0.000*	0.015±0.002*	0.028±0.003*	0.031±0.003	0.029±0.004

The results are presented as the average values ± SEM of 5 animals. * indicates a significant difference from the baseline (0 h) (one-way ANOVA with Tukey's post-test, $P < 0.05$).

Supplementary table S4 Acylcarnitine (AC) concentrations in heart during an oral glucose tolerance test.

	**Time after glucose administration**					
**nmol/g**	**0 h**	**0.5 h**	**1 h**	**2 h**	**4 h**	**6 h**
**C2**	184±26	225±17	280±42	247±31	272±19	197±14
**C3**	4.7±0.6	4.7±0.3	5.0±1.0	3.7±0.6	4.0±0.5	2.9±0.2*
**C4**	7.6±0.7	4.6±0.5*	7.0±2.0	6.9±1.6	8.4±1.1	5.8±0.7
**C5**	0.66±0.08	0.67±0.06	0.64±0.08	0.87±0.10	0.80±0.14	0.56±0.05
**C6**	1.5±0.1	0.7±0.1*	0.9±0.1*	1.1±0.3	2.4±0.4	1.5±0.3
**C8**	0.52±0.06	0.30±0.03*	0.33±0.02*	0.46±0.12	0.98±0.14*	0.55±0.13
**C10**	0.39±0.04	0.22±0.02*	0.30±0.04	0.42±0.10	0.95±0.20*	0.47±0.13
**C12**	0.58±0.06	0.22±0.02*	0.33±0.03*	0.51±0.13	1.12±0.26	0.61±0.20
**C14**	2.4±0.2	0.6±0.1*	1.1±0.2*	1.8±0.,5	4.0±1.0	2.4±0.9
**C16**	10.8±0.8	2.7±0.4*	6.4±1.4*	8.0±2.2	16.0±3.5	10.3±3.5
**C18:0**	4.8±0.5	2.0±0.2*	3.4±0.5	3.1±0.5*	6.4±0.8	4.7±1.1
**C18:1**	15.7±0.7	5.5±0.7*	10.2±2.1*	9.7±2.5*	18.4±3.9	12.2±3.9
**C18:2**	2.5±0.2	1.1±0.1*	1.5±0.3*	1.7±0.4	3.5±0.9	1.9±0.6

The results are presented as the average values ± SEM of 5 animals. * indicates a significant difference from the baseline (0 h) (one-way ANOVA with Tukey's post-test, $P < 0.05$).

Supplementary table S5 Acylcarnitine (AC) concentrations in skeletal muscles during an oral glucose tolerance test.

	**Time after glucose administration**					
**nmol/g**	**0 h**	**0.5 h**	**1 h**	**2 h**	**4 h**	**6 h**
**C2**	267±30	218±36	357±48	297±35	267±21	261±19
**C3**	7.4±0.9	10.6±1.4	7.0±0.5	5.2±0.6	3.8±0.3*	3.9±0.9*
**C4**	4.3±0.5	3.1±0.4	4.1±0.5	3.7±0.1	4.1±0.2	3.8±0.3
**C5**	0.33±0.01	0.77±0.18*	0.60±0.25	0.62±0.07*	0.33±0.02	0.27±0.02*
**C6**	0.24±0.02	0.35±0.11	0.64±0.16	0.66±0.10	0.79±0.05	0.61±0.12
**C8**	0.06±0.01	0.10±0.03	0.13±0.04	0.14±0.03*	0.18±0.02*	0.16±0.04*
**C10**	0.06±0.01	0.11±0.04	0.12±0.03	0.11±0.03	0.13±0.02*	0.13±0.03*
**C12**	0.07±0.01	0.12±0.03	0.18±0.07	0.16±0.03*	0.22±0.04*	0.20±0.04*
**C14**	0.32±0.03	0.51±0.15	0.87±0.35*	0.80±0.18*	1.05±0.24*	0.90±0.22*
**C16**	1.9±0.2	2.6±0.6	4.7±2.1	3.5±0.7*	4.2±0.9*	4.2±1.3
**C18:0**	0.7±0.1	1.0±0.2	1.4±0.4*	1.3±0.2*	1.4±0.2*	1.4±0.4
**C18:1**	4.4±0.5	4.9±1.0	7.9±3.3	5.7±0.7	9.0±1.5*	9.3±1.1*
**C18:2**	0.8±0.1	0.9±0.2	1.3±0.5	1.2±0.2	2.1±0.4*	2.1±0.3*

The results are presented as the average values ± SEM of 5 animals. * indicates a significant difference from the baseline (0 h) (one-way ANOVA with Tukey's post-test, $P < 0.05$).

Supplementary table S6 Acylcarnitine (AC) concentrations in liver during an oral glucose tolerance test.

	**Time after glucose administration**					
**nmol/g**	**0 h**	**0.5 h**	**1 h**	**2 h**	**4 h**	**6 h**
**C2**	58±14	68±6	45±4	45±7	47±2	39±3
**C3**	5.9±1.1	7.3±0.6	8.9±1.0	6.4±1.5	6.9±1.3	5.4±1.7
**C4**	2.0±0.6	4.6±0.4*	4.9±0.7*	3.3±1.0	2.5±0.5	2.6±0.9
**C5**	0.51±0.08	0.83±0.06*	0.99±0.11*	0.62±0.08	0.61±0.06	0.61±0.13
**C6**	0.18±0.03	0.44±0.07*	0.38±0.06*	0.24±0.05	0.31±0.05*	0.28±0.08
**C8**	0.07±0.01	0.15±0.02*	0.12±0.01*	0.08±0.01	0.13±0.02*	0.10±0.02
**C10**	0.04±0.01	0.06±0.01	0.06±0.01	0.05±0.01	0.11±0.02*	0.07±0.01
**C12**	0.03±0.00	0.06±0.01*	0.06±0.01*	0.05±0.01*	0.07±0.01*	0.06±0.01*
**C14**	0.09±0.01	0.17±0.02*	0.16±0.02*	0.18±0.02*	0.20±0.03*	0.19±0.02*
**C16**	1.2±0.2	2.0±0.2*	1.7±0.3	1.8±0.3	2.0±0.3	1.7±0.3
**C18:0**	0.7±0.1	0.7±0.1	0.8±0.1	0.8±0.1	1.0±0.2	0.9±0.1
**C18:1**	3.8±0.9	8.3±1.4*	6.1±1.4	7.1±2.5	5.5±1.2	4.7±1.2
**C18:2**	0.9±0.2	2.6±0.5*	1.6±0.4	1.9±0.7	1.9±0.5	1.3±0.3

The results are presented as the average values ± SEM of 5 animals. * indicates a significant difference from the baseline (0 h) (one-way ANOVA with Tukey's post-test, $P < 0.05$).
